# Effect of a plant-based diet on oxidative stress biomarkers in male footballers

**DOI:** 10.1038/s41598-024-54198-3

**Published:** 2024-02-14

**Authors:** Mahsa Zare, Niloofar Shoaei, Jahangir Karimian, Mehran Nouri, Sahar Zare, Kimia Leilami, Zainab Shateri, Parvin Sarbakhsh, Mohammad Hasan Eftekhari, Bahram Pourghassem Gargari

**Affiliations:** 1 Student Research Committee, Faculty of Nutrition and Food Science, Tabriz University of Medical Sciences, Tabriz, Iran; 2 Department of Community Nutrition, School of Nutrition and Food Science, Food Security Research Center, Isfahan University of Medical Sciences, Isfahan, Iran; 3 Department of General Courses, School of Management and Medical Information Sciences, Isfahan University of Medical Sciences, Isfahan, Iran; 4 Department of Community Nutrition, School of Nutrition and Food Science, Shiraz University of Medical Sciences, Shiraz, Iran; 5 Health Research Institute, Babol University of Medical Sciences, Babol, Iran; 6 Nursing Department, Eghlid Branch, Islamic Azad University, Eghlid, Iran; 7 Nutrition Research Center, Shiraz University of Medical Sciences, Shiraz, Iran; 8 Department of Nutrition and Biochemistry, School of Medicine, Ilam University of Medical Sciences, Ilam, Iran; 9 Department of Statistics and Epidemiology, School of Public Health, Tabriz University of Medical Sciences, Tabriz, Iran; 10 Department of Clinical Nutrition, School of Nutrition and Food Sciences, Shiraz University of Medical Sciences, Shiraz, Iran; 11 Nutrition Research Center, Department of Biochemistry and Diet Therapy, Faculty of Nutrition and Food Sciences, Tabriz University of Medical Sciences, Tabriz, Iran

**Keywords:** Biomarkers, Health care, Risk factors

## Abstract

Proper nutrition plays a vital role in modulating oxidative status. There is an increasing popularity of plant-based dietary patterns among athletes. Therefore, the present study aimed to determine the plant-based diet index (PDI) score among male footballers and their non-athlete controls, as well as its relationship with oxidative biomarkers by evaluating the urinary excretion of F_2alpha_-isoprostane (F_2a_-IP) and 8-hydroxy-2′-deoxyguanosine (8-OHdG). A group of footballers (n = 45) and a healthy non-athlete group (n = 45) were selected. The two groups were matched based on body mass index (BMI) and age. The mean (standard deviation (SD)) age of the subjects was 22.88 (2.41) years, and their BMI was 22.08 (1.35) kg/m^2^. Anthropometric indices were evaluated, and fasting morning urine samples were analyzed to measure oxidative biomarkers. The PDI, unhealthy plant-based diet index (uPDI), and healthy plant-based diet index (hPDI) were calculated using valid food frequency questionnaire data. Generalized estimating equation models were used for all analyses. Compared to the non-athlete group, the PDI score was significantly greater in the footballer group (*β* = 9.8; *P* < 0.001), while the differences between the two groups in uPDI and hPDI scores were not significant. Overall, footballers consumed more plant-based foods. By examining the relationship between dietary indices and oxidative biomarkers, only a negative association was observed between PDI score and F_2a_-IP level (*β* = −1.25; *P* = 0.03). Based on the results, footballers were more adherent to a plant-based diet than non-athletes. In addition, it seems that following plant-based diets (the higher PDI) may exert beneficial effects on lowering F_2a_-IP levels due to improving the body's antioxidant status.

## Introduction

Football is the most popular sport worldwide^1^. This endurance team sport relies on both anaerobic and aerobic energy pathways and includes highly energy-demanding efforts (sprints, jumps, high-intensity running, and direction change). Subsequently, these efforts can trigger the oxidative stress^2–5^.

Oxidative stress is characterized by a disturbance in the balance between the production of reactive oxygen species and their deactivation, leading to cellular deoxyribonucleic acid (DNA), lipid, and protein damage^6^. Various markers of oxidative damage exist, including urinary concentrations of 8-hydroxy-2′-deoxyguanosine (8-OHdG), a measure of oxidative DNA damage by hydroxyl radicals^6^, and F_2alpha_-isoprostane (F_2a_-IP), a measure of lipid oxidative damage caused by arachidonic acid peroxidation^7^, both of which are reliable^8,9^. It has been reported that the concentrations of 8-OHdG and F_2a_-IP in the urine have been associated with various parameters such as age, gender, body mass index (BMI), dietary antioxidant intake, smoking, and exercise^10^. Regarding the concentrations of 8-OHdG and F_2a_-IP in exercise, an increase in 8-OHdG and F_2a_-IP levels has been found immediately after exercise. At the same time, a decreasing trend has been observed at least two days after exercise^11–13^.

Nutrition plays an important role among athletes because of the association between the composition of the diet and sports success^14,15^. There is an increasing popularity of plant-based dietary patterns, especially vegan and semi-vegetarian, or flexitarian diets, among athletes^16^.

Plant-based diets are classified based on the relative proportions of plant- and animal-based foods^17,18^, meaning that these dietary patterns emphasize higher consumption of plant-based food groups, such as whole grains, nuts, legumes, fruits, seeds, vegetables, and less consumption of animal-based foods^19,20^. It has been demonstrated that dietary patterns with a high content of plant food products may have a favorable effect on the oxidative status and defend against the uncontrollable levels of reactive oxygen species^21,22^.

Given the body of evidence that supports the valuable role of nutritional strategies in the health and performance of footballers^23^, as well as the evidence of the benefits of plant-based diets on athletes^24,25^, it seems that the plant-based diet index (PDI) can be useful in footballers as a reliable tool to determine the adherence to a plant-based dietary pattern^26^.

To our knowledge, no research has been conducted on the determination of a plant-based diet and its relationship with urinary oxidative biomarkers in footballers. Also, based on the evidence, it can be hypothesized that adherence to a plant-based dietary pattern is different between footballers and non-athletes. In addition, there is a relationship between plant-based diets and oxidative biomarkers. Therefore, the present study aimed to investigate three dietary indices (PDI, unhealthy plant-based diet index (uPDI), and healthy plant-based diet index (hPDI)) in male footballers and their non-athlete controls. Also, we assessed the associations of the aforementioned dietary indices with urinary excretion of F_2a_-IP and 8-OHdG.

## Methods

### Study design, participants, and sampling

This descriptive-analytical study is part of a research project, some of the findings of which have already been published^27,28^. A total of 90 males (45 footballers and 45 healthy non-athlete controls) aged 20–30 with a BMI of 20–25 kg/m^2^ from Shiraz, Iran, participated in the study. Participant recruitment and data collection were took place between April 2022 and July 2022.

Eligible footballers included the following criteria:

A. Football experience for the last two years and a training protocol of 3–4 sessions/week and 1.5–2 h/session.
B. Metabolic equivalent of task (MET) more than 3000 (min/week).
C. No change in eating behaviors.
D. Not consuming alcohol and smoking.
E. Not taking antioxidant supplements.

Eligible non-athletes met the following criteria: matched to footballers based on BMI and age, 600 < MET < 3000 (min/week), and items #C-E (items listed above for footballers).

The following items were considered as the exclusion criteria for both groups:

1. The presence of any clinical disease based on the subject's self-reported medical information.
2. Taking drugs that change the oxidant-antioxidant balance and non-steroidal anti-inflammatory drugs (NSAIDs).
3. Those who completed less than 90% of the food frequency questionnaire (FFQ) items.

**Figure 1:**
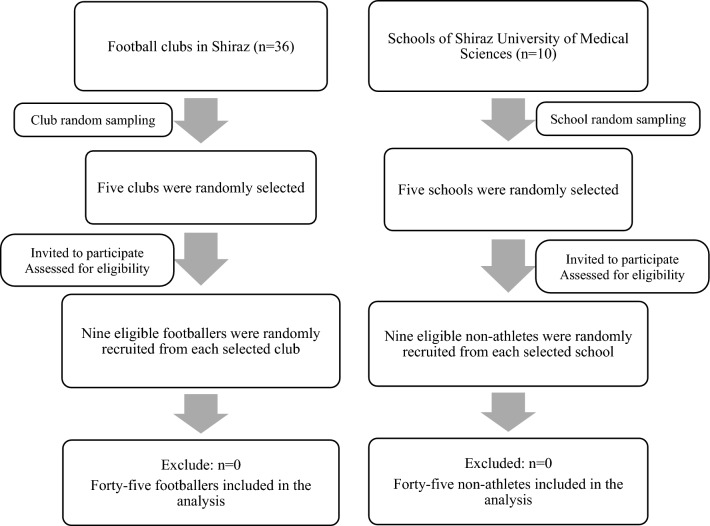
Flow chart of the participants' recruitment.

All participants were included in the study using cluster sampling method from 36 Shiraz football clubs and ten schools of Shiraz University of Medical Sciences. In the first stage, five clubs and five schools were randomly selected. At the next step, 45 footballers (nine persons from each selected club) and 45 non-athletes (nine persons from each selected school) who met the inclusion criteria were randomly recruited for the athlete and non-athlete groups, respectively. The flow chart of the participants' recruitment is illustrated in Fig. 1.

### Measurements

All participants were interviewed by a skilled dietitian to obtain data on general nutrition, medical history, physical activity, and food intake, and then anthropometric measurements and urine sampling were performed.

Height was measured by a stadiometer with an accuracy of 0.1 cm (Seca 222, Hamburg, Germany), and weight was measured by a digital scale with an accuracy of 0.1 kg (Seca 813, Hamburg, Germany) without shoes and with light clothes. BMI was calculated as weight/height^2^ (kg/m^2^). Physical activity was expressed as MET-min per week and was extracted from the International Physical Activity Questionnaire (IPAQ)^29^.

### Dietary assessment and calculation of PDI

Participants' food intakes over the last year were extracted by the interviewer through a 168-item semi-quantitative FFQ^30^. Respondents were asked to report their usual consumption of each item (per day, week, month, or year), then were converted to grams per day based on household measures^31^. Finally, the Nutritionist IV software modified for Iranian foods (First Databank, San Bruno, CA, USA) was utilized to obtain each food item-related data.

Based on the data extracted from FFQ, the PDI was calculated using the method proposed in Satija et al. study^32^. Three total indices of PDI, uPDI, and hPDI were calculated. Based on the nutrient similarity of the 18 food groups, they were classified into three larger groups, which included:

A. Healthy plant foods (whole grains, nuts, legumes, fruits, vegetables, vegetable oils, and tea/coffee).
B. Less healthy plant foods (refined grains, fruit juices, potatoes, sugar-sweetened beverages, and sweets/desserts).
C. Animal foods (dairy, egg, meat, animal fat, miscellaneous animal-based foods, and fish/seafood).

Examples of foods in each food group are listed in Table 1. Each food item was rated into quintiles of consumption. Subsequently, they received positive or inverse scores. For positive scores, the highest and lowest quintiles of consumption received a score of 5 and 1, respectively, while for inverse scores, the scoring pattern was reversed. To calculate the PDI, positive scores were applied to foods in groups A and B, while inverse scores were applied to group C. To calculate the hPDI, group A was allocated a positive score, while groups B and C were allocated inverse scores. To calculate the uPDI, group B received a positive score, while groups A and C received inverse scores. The scores of all food groups were summed to establish the indices, with a range of 18–180. A higher score in each of the three indicators indicates greater adherence to that food pattern. Notably, first, in calculating the mentioned dietary scores, energy-adjusted values of each food item were calculated using the residual method^33^.

**Table 1:** Examples of food items in 18 food groups.

**Healthy** **plant food groups**	
Whole grains	Dark bread, oatmeal, cereal, other grains
Nuts	Peanut butter, nuts
Legumes	Beans or lentils, soybeans, lima beans or peas, string beans
Fruits	Watermelon, melon, cantaloupe, grapefruit, oranges, apples, pears, strawberries, tangerine, lemon, pomegranate, kiwi, persimmon, cherry, peaches, apricots or plums, date, fresh and dried berries, green olives pineapple, raisins or grapes, fig, bananas nectarine
Vegetables	Tomatoes, tomato sauce, mushrooms, onion, cauliflower, broccoli, carrots, mixed vegetables, cabbage, winter or yellow squash, celery, zucchini, beets, eggplant, cooked or raw spinach, garlic, leaf lettuce
Vegetable oils	Vegetable oil used for cooking, olive oil
Tea and coffee	Coffee, tea

**Less healthy plant food groups**	
Refined grains	White rice, macaroni, white bread, vermicelli, muffins or biscuits, crackers
Fruit juices	Apple juice, grapefruit juice, orange juice, other fruit juice
Potatoes	Baked potatoes, potato chips, French fries
Sugar sweetened beverages	Colas with sugar, canned fruits, fruit drinks with sugar
Sweets and desserts	Candy bars, chocolates, cake, cookies, sweet roll, and pie, jellies, preserves, jams, syrup, honey

**Animal food groups**	
Dairy	Whole milk, low fat milk, ice cream, cream, yogurt, dough, cheese and cream cheese
Egg	Eggs
Meat	Chicken or turkey, hamburger, hot dogs, kielbasa, liver, processed meats, beef or lamb mixed dish
Animal fat	Butter or lard used for cooking, butter added to food
Miscellaneous animal-based foods	Mayonnaise sauce, pizza
Fish or seafood	Shrimp, canned tuna, other fish

### Laboratory investigation

Morning urine of all subjects was collected from 8–10 am after a 12-h fast (for footballers, 72 h after the last football training). The levels of 8-OHdG were measured by a competitive enzyme-linked immunosorbent assay (ELISA) using a commercial kit (8-OHdG kit, Abbexa, United Kingdom; Catalog No: abx150312). F_2a_-IP levels were also measured by a competitive ELISA using a commercial kit (8-epi PGF2a kit, Abbexa, United Kingdom; Catalog No: abx150311). Then, the obtained values were standardized based on the amount of creatinine in urine. Creatine was measured using a spectrophotometry test kit (Pars Azmoon, Tehran, Iran).

### Sample size estimation

The sample size was determined based on the mean difference in urinary 8-OHdG concentration between athlete and non-athlete groups provided by Rahimi et al.'s study^34^, using G*power software. According to the paired design of the study and the existing correlation between the studied groups, the relevant formula of such studies was used by considering α = 0.05 (95% confidence), β = 0.8, and a correlation of 0.15 between the 8-OHdG levels in the studied groups. As a result, 43 people were needed for each group of athletes and non-athletes. To compensate for about 5% of the sample dropout, 45 persons were recruited for each group.

### Statistical analysis

The Kolmogorov–Smirnov test was utilized to check the normality distribution of the variables. Normal variables were reported as mean ± standard deviation (SD), and data with non-normal distribution as median (25th–75th percentile). The generalized estimating equation (GEE) analysis was performed for all data analyses by considering an exchangeable correlation structure and identity link function. The GEE approach was applied to compare the mean values of general characteristics and food intakes in study groups and to examine the differences in PDI, hPDI, and uPDI scores between the footballer and non-athlete groups. To evaluate the association between dietary indices (PDI, hPDI, and uPDI) and oxidative biomarkers (8-OHdG and F_2a_-IP levels), a linear regression analysis was performed using the GEE in two models, model 1: crude, and model 2: adjusted for MET. All the statistical analyses were performed using the Statistical Package for Social Sciences (SPSS) (SPSS Inc., Chicago, IL, USA) with a significance level of 0.05 for all tests.

### Ethics approval and consent to participate

This study was in accordance with the principles of the Declaration of Helsinki and the ethical considerations of the Ethics Committee of Tabriz University of Medical Sciences (Approval Number: IR.TBZMED.REC.1399.1009). All participants signed a written informed consent.

## Results

### Participants’ general characteristics and oxidative biomarkers

Table 2 represents the subject’s general characteristics. The mean (SD) age of the subjects was 22.88 (2.41) years, and their BMI was 22.08 (1.35) kg/m^2^. Compared to the non-athlete group, the footballer group was significantly higher in MET (*P* < 0.001) and PDI score (*P* < 0.001). The differences between the two study groups regarding hPDI and uPDI scores were not statistically significant.

**Table 2:** The general characteristics and plant-based dietary scores of the participants.

Variables	Footballers (n = 45)	Non-athletes (n = 45)	All participants (n = 90)	*P*-value*
BMI (kg/m^2^)	22.06 ± 1.34	22.09 ± 1.37	22.08 ± 1.35	0.90
Age (years)	22.89 ± 2.42	22.87 ± 2.42	22.88 ± 2.41	0.99
MET (min/week)	3760.95 ± 542.98	972.76 ± 375.69	2366.86 ± 1476.77	** < 0.001**
PDI	104.15 ± 14.74	94.26 ± 10.48	99.15 ± 13.63	** < 0.001**
hPDI	100.28 ± 12.35	97.84 ± 13.17	99.07 ± 12.75	0.41
uPDI	97.56 ± 11.12	100.20 ± 16.71	98.89 ± 14.21	0.37

Values are expressed as mean ± SD.

Significant p-values (< 0.05) are in bold.

*BMI* body mass index, *MET* metabolic equivalent of task, *PDI* plant-based diet index, *hPDI* healthy plant-based diet index, *uPDI* unhealthy plant-based diet index.

*P-value is based on the GEE model with identity link function and exchangeable correlation structure.

**Figure 2:**
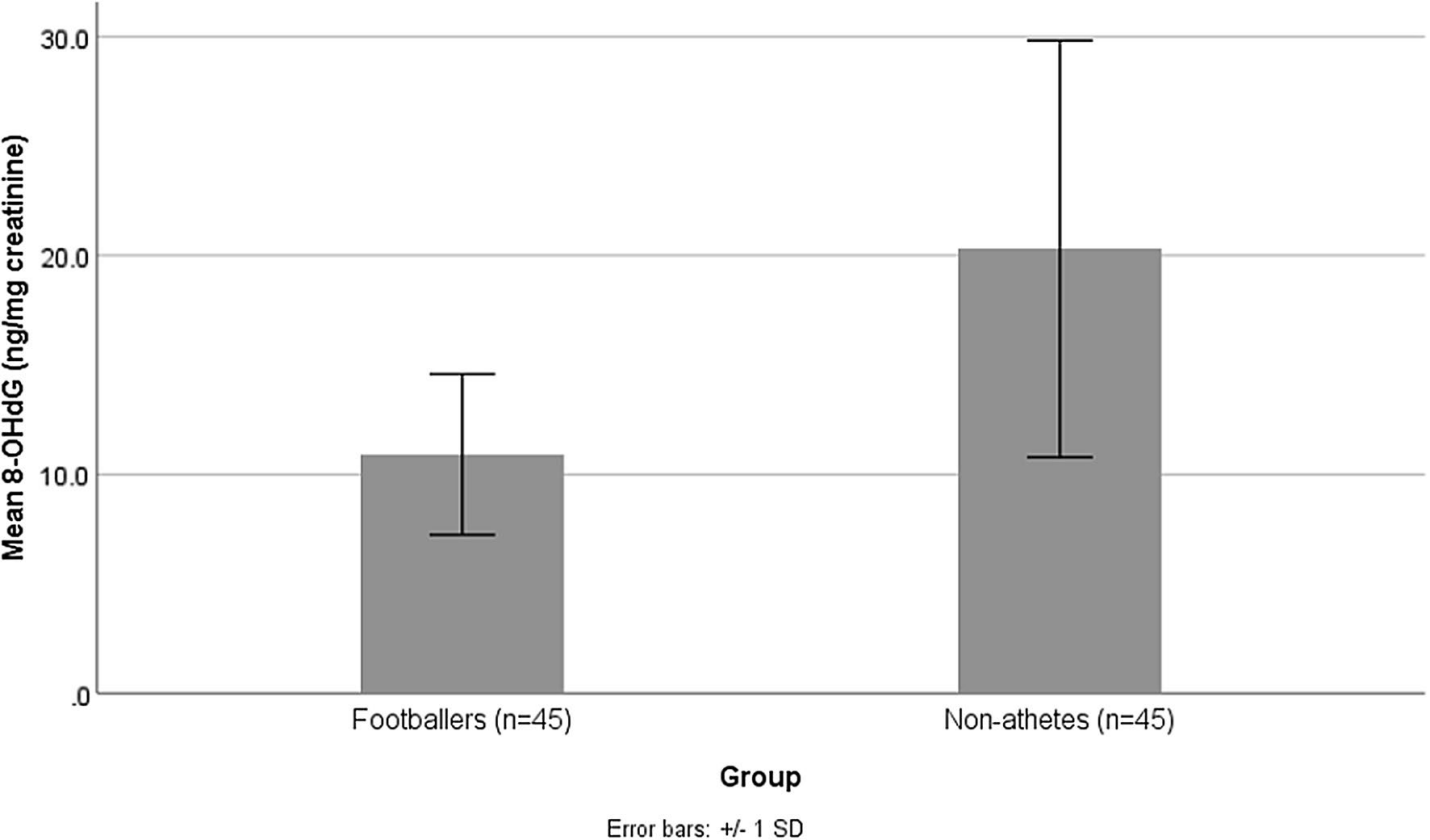
Mean concentrations of urinary 8-OHdG (ng/mg creatinine) in the footballer and non-athlete groups. *8-OHdG* 8-hydroxy-2′-deoxyguanosine. *P*-value < **0.001**. P-value < 0.05 considered as statistically significant.

**Figure 3:**
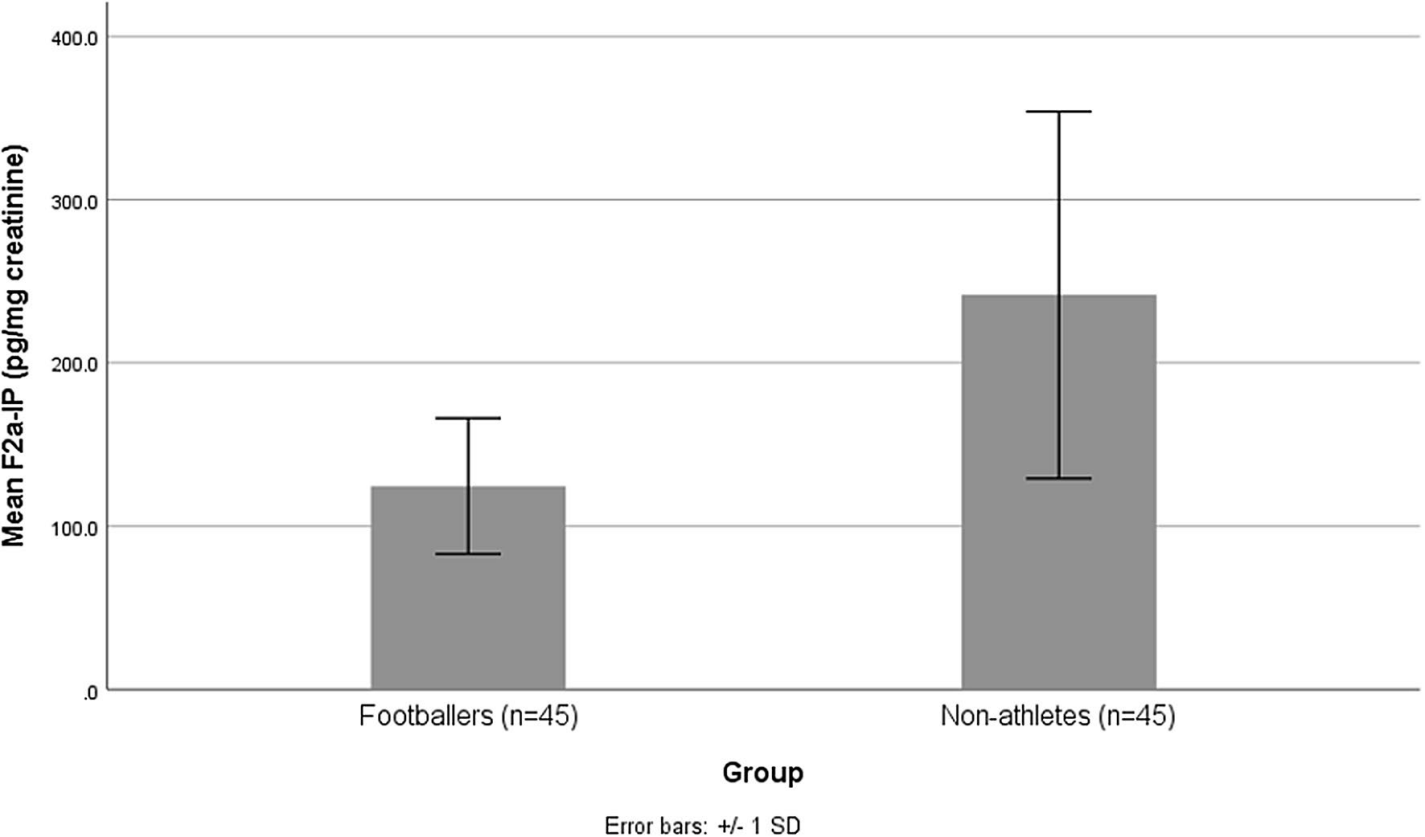
Mean concentrations of urinary F_2a_-IP (pg/mg creatinine) in the footballer and non-athlete groups. *F*_*2a*_*-IP* F_2alpha_-isoprostane. *P*-value < **0.001**. *P*-value < 0.05 considered as statistically significant.

The mean 8-OHdG concentration of participants is presented in Fig. 2. Footballer group was significantly lower in 8-OHdG (*P* < 0.001). Also, Fig. 3 shows the mean F_2a_-IP concentration of participants. F_2a_-IP (*P* < 0.001) concentration was lower in the footballer group than in the control group.

### Dietary intakes

Table 3 shows the intake of 18 food groups. In terms of the examined food groups, refined grains (*P* = 0.009), nuts (*P* = 0.01), fruits (*P* = 0.009), fruit juice (*P* < 0.001), vegetables (*P* = 0.01), potatoes (*P* = 0.02), tea and coffee (*P* = 0.002), dairy (*P* < 0.001), egg (*P* < 0.001), and fish or seafood (*P* < 0.001) were significantly higher in footballer group. In contrast, vegetable oils (*P* = 0.001), meats (*P* < 0.001), animal fat (*P* < 0.001), and miscellaneous animal-based foods (*P* = 0.004) were significantly lower in the footballer group than in the control group.

**Table 3:** Dietary intakes of the participants.

Variables	Footballers (n = 45)	Non-athletes (n = 45)	All participants (n = 90)	*P*-value*
Whole grains (g/day)	56.56 (34.14–88.76)	31.50 (25.00–68.82)	43.67 (27.06–75.58)	0.08
Refined grains (g/day)	461.54 ± 87.21	418.02 ± 88.95	439.78 ± 90.28	**0.009**
Nuts (g/day)	15.60 (10.93–24.50)	13.13 (10.28–17.36)	14.91 (11.14–20.72)	**0.01**
Legumes (g/day)	71.03 ± 13.30	67.19 ± 16.99	69.11 ± 15.29	0.19
Fruits (g/day)	266.88 ± 124.21	206.71 ± 100.78	236.79 ± 116.46	**0.009**
Fruit juice (g/day)	22.60 (17.30–35.70)	6.20 (3.90–14.50)	16.80 (3.90–24.00)	** < 0.001**
Vegetables (g/day)	327.04 ± 112.67	275.88 ± 97.26	301.46 ± 107.77	**0.01**
Vegetable oils (g/day)	26.28 ± 9.15	32.64 ± 8.16	29.46 ± 9.19	**0.001**
Potatoes (g/day)	41.29 ± 11.52	35.91 ± 11.05	38.60 ± 11.55	**0.02**
Tea and coffee (g/day)	200.00 (100.00–357.85)	108.00 (62.00–215.00)	144.10 (78.75–261.25)	**0.002**
Sugar-sweetened beverages (g/day)	53.90 (29.86–92.75)	41.60 (25.75–84.80)	47.00 (26.97–90.82)	0.06
Sweets and desserts (g/day)	39.96 ± 13.57	41.43 ± 18.00	40.70 ± 15.87	0.68
Dairy (g/day)	271.70 ± 105.58	192.62 ± 80.25	232.16 ± 101.37	** < 0.001**
Egg (g/day)	45.80 (36.25–78.20)	30.00 (20.00–36.60)	36.60 (25.00–53.50)	** < 0.001**
Meats (g/day)	44.24 ± 10.97	55.50 ± 16.61	49.87 ± 15.10	** < 0.001**
Animal fat (g/day)	5.70 (3.08–7.83)	9.00 (4.92–14.25)	7.20 (4.07–10.37)	** < 0.001**
Miscellaneous animal-based foods (g/day)	6.59 (3.55–9.90)	8.30 (4.40–16.15)	7.69 (3.97–12.80)	**0.004**
Fish or seafood (g/day)	9.80 (6.15–15.90)	4.50 (2.26–8.32)	6.97 (2.97–11.95)	** < 0.001**

Values are expressed as mean ± SD or median (25th–75th percentile).

Significant p-values (< 0.05) are in bold.

*P-value is based on the GEE model with identity link function and exchangeable correlation structure.

### Comparison of dietary indices

A comparison of PDI, uPDI, and hPDI differences between the two groups is presented in Table 4. Based on these results, the PDI score of the footballer group was significantly higher than the non-athlete group (*β* = 9.8; *P* < 0.001). However, the two groups had no differences in hPDI and uPDI scores.

**Table 4:** The comparison of PDI, hPDI, and uPDI scores between footballer and non-athlete groups.

Variables	*B*	SE	95% Wald confidence interval (lower , upper)	*P*-value*
PDI				
Non-athletes	Reference	–	–	–
Footballers	9.80	2.58	(4.81 , 14.93)	** < 0.001**
hPDI				
Non-athletes	Reference	–	–	–
Footballers	2.45	3.02	(− 3.47 , 8.38)	0.41
uPDI				
Non-athletes	Reference	–	–	–
Footballers	− 2.63	2.96	(− 8.44 , 3.18)	0.37

Significant p-value (< 0.05) is in bold.

*B* regression coefficient, *SE* standard error, *PDI* plant-based diet index, *hPDI* healthy plant-based diet index, *uPDI* unhealthy plant-based diet index.

*P-value is based on the GEE model with identity link function and exchangeable correlation structure.

### Association between dietary indices and oxidative biomarkers

The results of linear regression analysis for the association of PDI, hPDI, and uPDI scores with 8-OHdG level are presented in Table 5. No statistically significant relationship was observed in any of the three indicators.

**Table 5:** The association between PDI, hPDI, and uPDI scores with 8-OHdG level in all participants.

Variables	*B*	SE	95% Wald confidence interval (lower , upper	*P*-value*
PDI				
Model 1	− 0.09	0.04	(− 0.18, 0.004)	0.05
Model 2	− 0.08	0.04	(− 0.18 , 0.007)	0.07
hPDI				
Model 1	− 0.04	0.07	(− 0.18, 0.09)	0.49
Model 2	–0.03	0.06	(− 0.17 , 0.09)	0.57
uPDI				
Model 1	0.04	0.05	(− 0.07, 0.15)	0.48
Model 2	0.002	0.06	(− 0.12 , 0.12)	0.97

Model 1: Crude, Model 2: Adjusted for MET.

*B* regression coefficient, *SE* standard error, *PDI* plant-based diet index, *hPDI* healthy plant-based diet index, *uPDI* unhealthy plant-based diet index.

*P-value is based on linear regression analysis using the GEE model (with identity link function and exchangeable correlation structure).

Regarding the association of PDI, hPDI, and uPDI scores with F_2a_-IP level, linear regression findings in Table 6 indicated that only a significant negative association was found between PDI score and F_2a_-IP level. With an increase of one unit of PDI, F_2a_-IP decreased by 1.25 (*P* = 0.03).

**Table 6:** The association between PDI, hPDI, and uPDI scores with F_2a_-IP level in all participants.

Variables	*B*	SE	95% Wald confidence interval (lower , upper)	*P*-value*
PDI				
Model 1	− 1.33	0.59	(− 2.49, − 0.16)	**0.02**
Model 2	− 1.25	0.58	(− 2.40, − 0.11)	**0.03**
hPDI				
Model 1	− 0.62	0.83	(− 2.25, 1,00)	0.44
Model 2	− 0.43	0.81	(− 2.02 , 1.16)	0.59
uPDI				
Model 1	0.63	0.82	(− 0.98, 2.25)	0.44
Model 2	0.19	0.87	(− 1.52 , 1.90)	0.82

Model 1: Crude, Model 2: Adjusted for MET.

Significant p-values (< 0.05) are in bold.

*B* regression coefficient, *SE* standard error, *PDI* plant-based diet index, *hPDI* healthy plant-based diet index, *uPDI* unhealthy plant-based diet index.

*P-value is based on linear regression analysis using the GEE model (with identity link function and exchangeable correlation structure).

## Discussion

The current study aimed to assess plant-based dietary indices in male footballers and healthy non-athlete controls and their associations with urinary oxidative biomarkers. The findings indicated that PDI was higher in footballers. Also, a negative association between PDI and F_2a_-IP was shown in all participants, and others did not show a statistically significant relationship.

The growing popularity of plant-based dietary patterns exists among athletes not only for the health advantages but also for the enhancement of athletic performance^35^. Plant-based dietary patterns may have the following effects: role in cardiovascular health, leaner body mass, facilitated glycogen storage, delayed fatigue, reduced oxidative stress, reduced inflammation, reduced blood viscosity, and increased tissue oxygenation^24,25^.

The current study showed that the footballer group had significantly higher PDI scores than the non-athlete group. However, no significant difference was shown in hPDI and uPDI scores between the two groups. Wirnitzer et al.^36^ stated that exercise alone is not sufficient for health. For this purpose, a healthy diet—preferably a complete vegan diet—is essential. Therefore, the integration of a healthy diet and a regular exercise program into the athletes' daily schedule can provide the most promising intervention to optimize their health^36^.

In the present study, footballers were more adherent to PDI than non-athletes, and also, in terms of PDI components, the intake of healthy plant-based food groups such as nuts, fruits, and vegetables was significantly higher in the footballers. Vegetables, whole grains, fruits, and beans are high in minerals, vitamins, and fiber, low in saturated fatty acids, and devoid of cholesterol^24^. In addition, footballers' total fat and animal fat intake was lower. Hinton et al.^37^ illustrated that more dietary fat intake could be related to cognitive problems in former football players. Therefore, they suggested encouraging football players to adhere to healthy dietary habits. Also, Fuhrman et al.'s study^38^ recommended that vegan and non-vegan athletes consume more beans, nuts, seeds, greens, whole grains, and other colorful plant-based products to maximize their recovery.

While several studies have shown that plant-based diets may be beneficial for athletes, some studies have stated that a vegetarian diet is not superior to an omnivorous diet in promoting health^39,40^. Consequently, following a plant-based diet may be a healthy option for athletes such as footballers. It is noteworthy that in any diet, nutrient adequacy is a critical consideration^24^. Therefore, any athlete who follows a plant-based diet should be supervised by a registered dietitian with a background in sports nutrition to ensure optimal nutrient and energy intake^39^.

We found a significant and negative association between PDI and F_2a_-IP in all participants. Although the number of athletes following plant-based diets is increasing, their relationship with oxidative stress is poorly understood, particularly in footballers. A systematic review study by Aleksandrova et al.^41^ revealed that diets high in fruits and vegetables, the Mediterranean diet, and the Dietary Approaches to Stop Hypertension (DASH) diet could lead to lower levels of F_2a_-IP. Further, Dietrich et al.^21^ observed vegans had lower excretion levels of F_2a_-IP than omnivores. Also, there was an inverse correlation between vegan diet and oxidative status measured by malondialdehyde, 8-OHdG, and F_2a_-IP. A study by Griffiths et al.^42^ indicated that more adherence to critical points of the Mediterranean diet, such as more consumption of vegetables, can reduce oxidative stress and inflammation.

It seems that the effect of plant-based diets on antioxidant properties is due to the high concentration of polyphenols and antioxidants present in these food patterns^35,43^. Tomey et al.^44^ reported an inverse relationship between three vegetable components (lutein, beta-carotene, and lycopene) and F_2a_-IP concentration in middle-aged women. The chemical structure of the mentioned molecules can lead to decreased peroxidation due to double bonds on polyene chains, which play a role in oxidation/reduction reactions^44^. Likewise, Block et al.^45^ revealed a negative correlation between beta-carotene and plasma levels of F_2a_-IP in healthy adults.

In addition to the compounds mentioned, other dietary factors such as dietary fat can also be associated with F_2a_-IP. Dietary fats may directly or indirectly affect the levels of F_2a_-IP, because (1) they are the main substrates of F_2a_-IP production, (2) they influence the plasma concentrations of F_2a_-IP transporters, and 3) they alter the tissue’s fatty acid composition^46^. Although the precise effect of dietary fats on F_2a_-IP levels in different populations still requires further investigation, it has been indicated that certain fatty acids, such as trans-fatty acids (TFA), may increase F_2a_-IP excretion in healthy adult individuals^46–48^. TFA can be incorporated into the cellular membrane’s phospholipids and reduce cell membrane fluidity through the displacement of cis-polyunsaturated fatty acids, which leads to more activity of free radicals in the phospholipids and the exacerbation of oxidative damage^49^. Since TFA is less present in plant-based food groups than in animal-based food groups^50^, it is possible that the higher the PDI, the lower the TFA intake, which may lead to less F_2a_-IP formation. Tomey et al.’s cohort study^44^ indicated a positive association between TFA intake and urine F_2a_-IP concentration in women. In addition, in other studies in healthy men and women, TFA increased urinary F_2a_-IP levels^51,52^. Furthermore, in vivo studies have confirmed that higher TFA intake is associated with higher urinary F_2a_-IP levels^53^. Altogether, it is likely that by consuming more plant-based foods, the body's antioxidant status is improved, and then the formation of F_2a_-IP is reduced.

As a strength of the study, to our knowledge, this is the first study to evaluate PDI and its relationship with urinary 8-OHdG and F_2a_-IP concentrations in footballers. Also, a valid and reliable FFQ was applied to assess food intake. However, this study had some limitations. Although FFQ and IPAQ questionnaires are useful tools for collecting information on usual dietary intake and physical activity, respectively, the use of FFQ and IPAQ may cause potential misreporting bias due to participants' memory errors. Another limitation was the potential for higher nutritional knowledge of the control participants due to being medical university students. The descriptive nature of the study possesses limitations; for example, it is impossible to infer the mechanisms of the results due to the cross-sectional design of this research. F_2a_-IP levels categorized as < 0.86 ng/mg creatinine and ≥ 0.86 ng/mg creatinine are considered low and high, respectively^54^. In our study, F_2a_-IP levels were classified as low in all participants (0.12 ng/mg creatinine in the footballer group and 0.24 ng/mg creatinine in the non-athlete group). Although even at this level, a relationship between plant-based diet score and oxidative biomarker was observed, further research is needed to determine whether this relationship exists at different levels of oxidative stress. Additionally, future research on other populations of athletes and non-athletes should be conducted to generalize the findings.

## Conclusions

The current study contributes to expanding our knowledge about plant-based diets and oxidative status. The findings revealed that footballers had greater PDI scores compared to non-athletes. Also, a negative association between PDI score and F_2a_-IP levels was observed in all participants. Altogether, it is likely that more adherence to dietary patterns with a high content of plant-based foods and, subsequently, more antioxidant-rich foods may help control oxidative status in footballers and physically active persons by reducing the formation of F_2a_-IP.

## Data Availability

Data are available through a reasonable request from the corresponding author.
